# Editorial: Nutrition in pancreatic diseases: the role of nutritional status and nutrition therapy in the management of pancreatitis and pancreatic cancer

**DOI:** 10.3389/fnut.2025.1753147

**Published:** 2026-01-07

**Authors:** Mats L. Wiese, Teresa Perra, Alberto Porcu

**Affiliations:** 1 Department of Food, Nutrition, Facilities, FH Münster University of Applied Sciences, Münster, Germany; 2 Azienda Ospedaliero Universitaria di Sassari, Sassari, Italy

**Keywords:** pancreas, pancreatitis, pancreatic cancer, malnutrition, sarcopenia, muscle loss, nutrition therapy, supplementation

The pancreas plays a pivotal physiological role in nutrient digestion and assimilation as well as metabolic control. Hence, it is not surprising that a close link between pancreatic disorders and nutritional status can be observed. Along the continuum of pancreatic diseases, an ambiguous relation with nutritional status exists (1). While obesity is a known risk factor for severe acute pancreatitis and pancreatic cancer, inflammation as well as an impaired exocrine and endocrine pancreatic function put patients at an increased risk of malnutrition, especially muscle loss. Because both excess body weight and disease-related malnutrition are associated with increased morbidity and mortality, adequate nutritional management of patients with pancreatic diseases is of key importance. However, there is still limited knowledge on the specific effects of nutritional status on disease outcome and the optimal modalities of nutrition therapy along the continuum of pancreatitis and pancreatic cancer. In this Research Topic, we compile research investigating the role of nutritional status and optimized nutritional management in pancreatic diseases.

Regarding acute pancreatitis, Chen et al. identify total cholesterol and triglyceride levels as nutrition-related risk factors that independently predict risk of acute pancreatitis in patients with diabetic ketoacidosis. In another work, Wang et al. studied predictive factors of disease severity in patients with hypertriglyceridemia-associated acute pancreatitis. Besides systemic immunoinflammatory index, the authors also find nutritional risk index and triglyceride-glucose index to predict severe acute pancreatitis. Notably, the combination of all three indices showed higher predictive value than any individual index. This finding supports the relevance of patients' nutritional and metabolic status in this form of acute pancreatitis. The complex relation of metabolic and nutritional factors with outcome in this entity is also addressed by Ma et al. Based on mechanistic considerations, the authors discuss in their review current and future approaches toward precision nutritional management in hyperlipidemia-associated acute pancreatitis.

While the link between acute pancreatitis and pancreatic cancer is complex and bidirectional (2), both entities share prominent common nutritional risk factors, namely obesity and alcohol consumption (1, 2). The growing burden of pancreatic cancer is addressed in a work by Dong et al. who report global trends in pancreatic cancer prevalence and mortality in individuals 55 years or older using data from the global burden of disease study 2021. The authors not only show geographical heterogeneity in temporal trends. They further link the growing burden in high socio-demographic index countries to increased body mass index in these regions.

To this day, surgical resection remains the only curative treatment option for pancreatic cancer, with pancreatoduodenectomy being the most performed surgical procedure (3). Three works included in this Research Topic investigate the prognostic value of nutritional status in patients undergoing pancreatoduodenectomy. In a retrospective study, Li, Hu, Yan et al. looked at the association of phase angle obtained by bioelectrical impedance analysis, which presents a non-invasive and feasible bedside method to estimate body composition, with nutritional status and clinical outcome in patients with pancreatic cancer. Although phase angle reflected nutritional status, especially malnutrition risk, only limited significance for predicting post-operative complications was found in this work. By contrast, Cai et al. investigated the predictive value of body composition assessed by computed tomography regarding surgical outcome following pancreatoduodenectomy. They find that lower skeletal muscle index was associated with higher occurrence of post-operative complications. Nevertheless, as analyses were performed on a retrospective dataset and the method could be limited in terms of diagnosing sarcopenia and visceral obesity, future prospective studies are clearly warranted. Nutritional status also seems to be relevant in terms of post-operative survival. Based on their in-house follow-up database Tian et al. examined the predictive value of pre-operative nutritional risk assessed by Nutritional Risk Screening 2002, a screening tool widely used in clinical practice. They show that patients with pre-operative nutritional risk, i.e., Nutritional Risk Screening 2002 ≥3 points, had a significantly shorter survival time, even when models were adjusted for several other known prognostic parameters. Although also this work needs confirmation, it emphasizes the importance of malnutrition risk screening in patients undergoing pancreatic surgery (4). Moreover, Li, Hu, Sheng et al. in another retrospective analysis add to the ongoing debate on ideal modalities of nutrition support in patients after pancreatoduodenectomy (5). Employing propensity score matching they demonstrate that the combination of early enteral with supportive parenteral nutrition in patients with pre-operative malnutrition risk was associated with a significantly lower incidence of severe post-operative complications compared to administration of total parenteral nutrition. Finally, another study highlights that also in patients with unresectable pancreatic cancer nutritional and metabolic status may affect prognosis. In this work by Xu et al., the predictive value of three-dimensional computed tomography derived body composition and triglyceride glucose-body mass index for one-year overall survival was tested. Several computed tomography-derived indicators of body composition as well as triglyceride glucose-body mass index were found independently associated with survival. Therefore, the nomogram model developed by the authors combining these risk factors presents an interesting and potentially helpful approach to provide risk-adapted nutritional treatment in this patient group.

In summary, the works compiled in this Research Topic expand the current state of knowledge on the relevance of nutritional and metabolic status in pancreatic diseases. However, it is also revealed that many aspects still require further investigation. Notably, the role of nutritional status in chronic pancreatitis, an entity with high malnutrition prevalence (6, 7), remains poorly investigated. As recent evidence suggests that intensified nutritional therapy may also improve prognosis in malnourished patients with chronic pancreatitis (8), it is desirable that future research addresses nutritional aspects in this entity as well. Finally, sarcopenia is a common condition among patients affected by pancreatic cancer. The nutritional status seems to have a prognostic role in patients undergoing pancreatic surgery (9). It would be useful, especially in patients who should undergo biliary stent placement or neoadjuvant chemotherapy while awaiting surgery, to follow a pre-habilitation program, which includes increased nutritional intake as well as to increase muscle strength, and therefore the possibility of greater physical activity performance.
